# Design and protocol of a randomized assessor-blinded controlled trial evaluating an educational escape room for individual and team cardiopulmonary resuscitation skills among nursing students

**DOI:** 10.1186/s12912-026-05151-4

**Published:** 2026-07-29

**Authors:** Fatemeh Bakhshi, Amir Dehghani Ashkezari, Sara Heydari

**Affiliations:** 1 Research Center for Nursing and Midwifery Care, Comprehensive Research Institute for Maternal and Child Health, School of Nursing and Midwifery, Shahid Sadoughi University of Medical Sciences, Yazd, Iran; 2 Department of Nursing, School of Nursing and Midwifery, Shahid Sadoughi University of Medical Sciences, Yazd, Iran; 3 Department of Medical Education, Medical Education and Development Center, Shahid Sadoughi University of Medical Sciences, Bouali Ave., Safaeyeh, Yazd, 8916877443 Iran

**Keywords:** Cardiopulmonary resuscitation, Nursing students, Gamified learning, Advanced life support, Educational escape room, Team coordination

## Abstract

**Background:**

High-quality cardiopulmonary resuscitation (CPR) depends not only on technical competence but also on effective communication, role clarity, leadership, and coordinated team performance under pressure. However, conventional CPR instruction may not adequately reproduce the collaborative and time-sensitive demands of real resuscitation. Educational escape rooms (EERs), as simulation-based gamified learning strategies, may provide a more immersive and learner-centered approach to strengthening both cognitive and teamwork-related aspects of CPR training. This study protocol was developed to evaluate the effect of a team-based CPR educational escape room (CPR-EER) integrated into advanced life support (ALS) training for undergraduate nursing students.

**Methods:**

This study was designed as a two-arm, parallel-group, assessor-blinded randomized controlled trial with a 1:1 allocation ratio. Participants were seventh-semester undergraduate nursing students at Shahid Sadoughi University of Medical Sciences, Yazd, Iran. All participants first received a standardized 8-hour CPR refresher workshop to establish a common educational baseline. The intervention group then participated in a structured CPR-EER delivered in fixed six-member teams in a simulated in-hospital cardiac arrest environment, whereas the control group continued with the routine curriculum during the primary comparison phase and received the intervention after post-test assessment. The primary outcome was team coordination during CPR, assessed using a Team Objective Structured Clinical Examination (TOSCE). Secondary outcomes included role-specific performance, CPR knowledge, and learner satisfaction. Outcomes were assessed at baseline and one month after the intervention. Data analysis was prespecified using a per-protocol framework and distribution-appropriate statistical tests.

**Discussion:**

This protocol provides a methodologically explicit and pedagogically grounded model for evaluating a gamified, team-based CPR-EER training strategy in undergraduate nursing education. By aligning intervention design, educational objectives, and outcome assessment, it may contribute to the development of more immersive, competency-oriented, and reproducible approaches to resuscitation training.

**Trial registration:**

Iranian Registry of Clinical Trials (IRCT), registered on 16 October 2024, IRCT20240815062770N1. Available at: https://irct.behdasht.gov.ir/.

**Supplementary Information:**

The online version contains supplementary material available at https://doi.org/10.1186/s12912-026-05151-4.

## Background

Cardiopulmonary arrest remains a critical clinical emergency that requires rapid recognition, coordinated action, and timely cardiopulmonary resuscitation (CPR) to improve the chance of survival [1, 2]. Although advances in resuscitation science have informed CPR guidelines, survival after cardiopulmonary arrest remains poor, and delays in initiating appropriate interventions can markedly reduce patient outcomes [3]. In addition, global estimates indicate that cardiac arrest continues to account for a substantial burden of mortality each year [4].

The quality of CPR is influenced not only by technical proficiency, but also by communication, leadership, teamwork, prior experience, fatigue, and motivation during resuscitation events [5]. Nurses, as front-line health professionals, are central members of resuscitation teams and are expected to provide immediate, accurate, and skillful interventions in emergency situations such as cardiopulmonary arrest [6]. In practice, successful CPR depends on a coordinated team in which each member performs a defined role efficiently and in synchrony with others [7, 8]. However, previous studies have shown persistent weaknesses in CPR-team coordination, role allocation, and team management, suggesting the need for educational approaches that better prepare nursing students for real resuscitation settings [9].

BLS and ALS are core components of CPR education, and ALS training has been associated with improved resuscitation outcomes and higher survival to discharge in hospitalized patients [10, 11]. For nursing students, however, conventional instructional methods may not fully reproduce the pressure, unpredictability, and collaborative demands of real CPR team performance. Accordingly, educational strategies that strengthen active participation, role clarity, and team coordination may be particularly valuable [12, 13].

Gamification has recently been introduced as an active, learner-centered educational approach that incorporates game elements to enhance engagement, motivation, and learning outcomes in nursing education [14, 15]. Within this broader approach, the educational escape room (EER) is a structured team-based learning method in which learners solve clinical tasks, clues, and problems within a limited time to achieve a specific goal [16, 17]. Because EER activities require collaboration, task delegation, role assignment, and shared decision-making, they may be particularly suitable for CPR education, where coordinated team performance is essential [18, 19].

Therefore, this study was designed to evaluate the effect of an CPR educational escape room (CPR-EER), a simulation-based gamified learning format, on CPR-related knowledge, role-specific performance, team coordination, and learner satisfaction among nursing students.

### Hypothesis

The study hypothesis was that participation in a CPR-EER integrated into ALS training would improve nursing students’ CPR-related performance, particularly team coordination and role clarity, compared with the control condition, and would also increase satisfaction with the learning method.

### Main objective

The main objective of this study was to evaluate the effect of a CPR-EER on team coordination during CPR among nursing students.

### Specific objectives

- To assess the effect of the CPR-EER on nursing students’ CPR knowledge.
- To assess the effect of the CPR-EER on nursing students’ role-specific performance during CPR.
- To assess the effect of the CPR-EER on team coordination during CPR.
- To assess nursing students’ satisfaction with the educational method used for ALS training.

## Methods/design

### Study design and setting

**Fig. 1:**
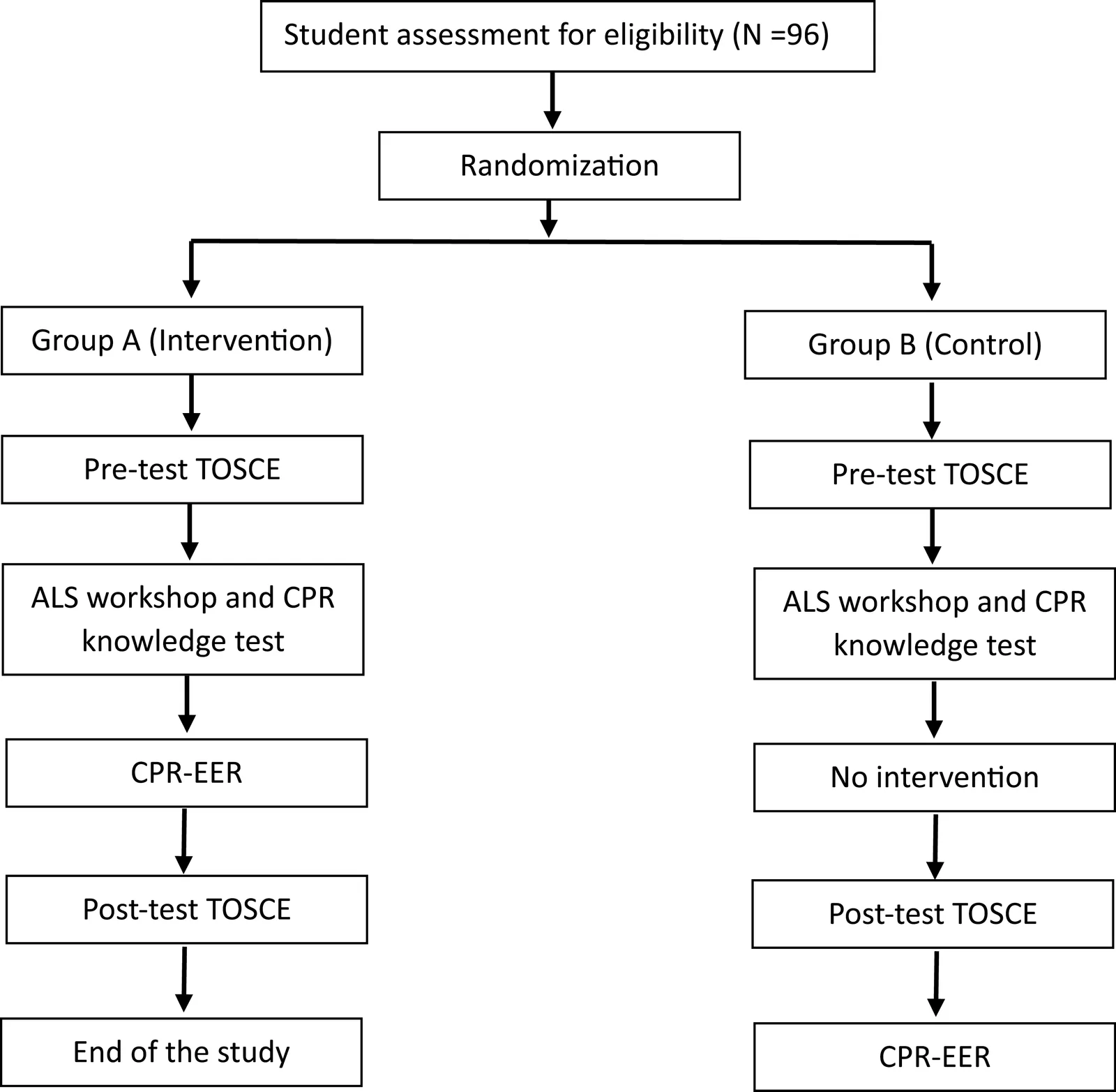
Flow diagram of participant recruitment, allocation, follow-up, and analysis in the CPR escape-room trial

This article reports the prespecified protocol of a two-arm, parallel-group, assessor-blinded randomized controlled trial with a 1:1 allocation ratio. The overall flow of participant recruitment, allocation, follow-up, and analysis is presented in Fig. 1. The trial was prospectively registered in the Iranian Registry of Clinical Trials (IRCT20240815062770N1), and ethical approval was obtained from the Ethics Committee of Shahid Sadoughi University of Medical Sciences, Yazd, Iran (Code: IR.SSU.REC.1403.068). The study was carried out at the School of Nursing and Midwifery of Shahid Sadoughi University of Medical Sciences, Yazd, Iran, during the 2024 academic year. The intervention and outcome assessments were implemented in the faculty’s clinical simulation environment, which was equipped to replicate a standard in-hospital cardiopulmonary resuscitation setting. This protocol is reported in accordance with the Standard Protocol Items: Recommendations for Interventional Trials (SPIRIT) guidance. Although trial procedures had been completed by the time the protocol manuscript underwent final editorial processing, the present article is confined to the study rationale, design, intervention procedures, outcome measures, and prespecified analytical framework and does not report trial results.

### Participants and eligibility criteria

Participants were undergraduate nursing students enrolled in the seventh semester of the nursing program. This academic level was selected because students had already completed relevant theoretical and clinical coursework related to emergency nursing and basic resuscitation, yet still represented a learner group in whom structured educational reinforcement of CPR team coordination was pedagogically appropriate. Students were eligible to participate if they were enrolled in the target semester, had previously completed the relevant CPR-related coursework, were willing to participate, and provided written informed consent. Students were excluded if they had a prior history of participation in a formal CPR-EER training program, declined to continue participation at any stage of the study, or were absent from any core educational or assessment session required for protocol completion. Because team-based performance constituted the primary outcome, students with incomplete participation in intervention delivery or outcome assessment were not eligible for inclusion in the final per-protocol dataset.

### Recruitment, sample size, and randomization

The target population comprised senior undergraduate nursing students enrolled in the seventh and eighth semesters. The sample size was calculated a priori using a two-sided significance level of 0.05 and a power of 80%, based on the effect estimates reported by Ma et al. [20]. Assuming a mean difference of 0.27 points between groups, with standard deviations of 0.43 and 0.45, the required sample size was estimated at 42 participants per group using the standard two-sample comparison formula. To account for an anticipated 10% attrition rate, the final target sample size was increased to 49 participants per group (98 participants in total). Because the primary outcome, team coordination in resuscitation, was evaluated at the team level using the TOSCE, we acknowledge the clustered structure of the outcome within fixed teams. Accordingly, the sample size and analysis plan were specified to account for team-based assessments, and the team structure was considered in interpreting the intervention effect. Recruitment was conducted through a public call distributed via the school’s extracurricular information channel. Eligible students were randomly allocated in a 1:1 ratio to the intervention and control groups using an online random sequence generator. Allocation was concealed by sequentially numbered, sealed opaque envelopes prepared by an independent person not involved in recruitment, intervention delivery, or outcome assessment. Any post-registration clarifications or amendments were limited to methodological details and did not alter the primary outcome, allocation ratio, or planned sample size. The participant pathway from enrollment to analysis is shown in Fig. 1.

### Masking

Because of the visible and experiential nature of the educational intervention, masking of participants and intervention facilitators was not feasible. However, outcome assessment was conducted under assessor-masked conditions. Examiners responsible for scoring the Team Objective Structured Clinical Examination (TOSCE) were not informed of group assignment, and efforts were made to preserve this masking throughout data collection. Statistical analyses were performed using coded datasets. Accordingly, this study is more appropriately characterized as an assessor-masked randomized controlled trial rather than a double-blind trial.

### Baseline and follow-up timeline

The study assessments were structured systematically across multiple consecutive phases, with the chronological schedule of enrollment, interventions, and evaluations summarized in Table 1. Prior to the initiation of any study procedures, all eligible participants completed a baseline demographic questionnaire, a 20-item electronic CPR knowledge test, and a pretest Team Objective Structured Clinical Examination (TOSCE) to measure baseline CPR knowledge, role-specific performance, and team coordination. Following these baseline evaluations, both groups participated in the standardized CPR refresher workshop. One week post-workshop, Group A underwent the CPR-EER intervention, while Group B continued with their standard curriculum (delayed-intervention control). One month after the intervention phase, both groups underwent the post-intervention assessments, which replicated the baseline evaluations (knowledge test and posttest TOSCE) and included a satisfaction questionnaire. Following the completion of all post-intervention evaluations, Group B was provided with the CPR-EER activity to ensure educational equity.

**Table 1:** Schedule of enrollment, interventions, and assessments. (adapted from the SPIRIT guidelines)

Study Period	Enrollment	Allocation	Post-Allocation (Intervention Phase)	Close-out
**Timepoint**	T_1_	T_0_	T_1_ (Week 1)	T_2_ (Month 1)
**Enrolment**				
Eligibility screen	✓			
Informed consent	✓			
Random allocation		✓		
**Interventions**				
CPR Refresher Workshop		✓		
CPR Escape-Room (Group A)			✓	
Standard Curriculum (Group B)			✓	
Delayed Escape-Room (Group B)*				✓
**Assessments**				
Demographic questionnaire	✓			
Electronic Knowledge Test	✓			✓
Pretest TOSCE	✓			
Posttest TOSCE				✓
Satisfaction Questionnaire				✓

*Note: Group B (control group) received the CPR escape-room intervention after the completion of all post-intervention assessments (T2) to maintain educational equity

### Common CPR refresher workshop

Before delivery of the group-specific trial condition, all enrolled participants attended a mandatory, standardized CPR refresher workshop designed to establish a common instructional foundation across both study arms. The workshop was incorporated to minimize variability in recent CPR exposure and to ensure that any subsequent between-group differences could be interpreted in relation to the additional escape-room training rather than unequal access to core resuscitation instruction. The workshop curriculum was developed in accordance with contemporary advanced life support (ALS) and cardiopulmonary resuscitation (CPR) recommendations, including the most recent guidance issued by the American Heart Association and the International Liaison Committee on Resuscitation [21–23]. Because the participants were seventh-semester undergraduate nursing students who had already completed the principal theoretical and clinical coursework in emergency and critical care nursing, the workshop was designed not to introduce foundational material for the first time, but rather to consolidate prior learning, refresh procedural knowledge, and reinforce structured team-based resuscitation performance [5, 24–26].

The training program was delivered in two consecutive 4-hour sessions, yielding a total of 8 h of contact time [27]. To facilitate active participation, deliberate practice, and close instructor supervision, participants attended the workshop in small cohorts of approximately 10 to 12 students [28]. The instructional strategy followed a blended educational format combining brief focused lectures, interactive case-based discussion, and simulation-based hands-on practice. Approximately 20% of contact time was allocated to concise didactic review, 30% to guided small-group discussion of clinical scenarios and decision points, and 50% to supervised practical training in the simulation laboratory using resuscitation manikins and standard CPR equipment [21].

The workshop content addressed the principal domains of in-hospital resuscitation competence relevant to undergraduate nursing practice. Initial teaching focused on rapid recognition of cardiopulmonary arrest, activation of the emergency response system, rescuer and patient safety, and structured primary assessment [29, 30]. Subsequent modules addressed high-quality chest compressions, compression–ventilation coordination, bag-valve-mask ventilation, airway adjunct use, rhythm recognition, manual defibrillation principles, and sequencing of critical actions during cardiac arrest management [31]. Participants also reviewed the distinction between shockable and non-shockable rhythms, including ventricular fibrillation, pulseless ventricular tachycardia, pulseless electrical activity, and asystole [32], as well as the indications and practical use of core resuscitation medications such as epinephrine and amiodarone [33].

In addition to technical skills, the workshop explicitly incorporated nontechnical competencies required for effective resuscitation teamwork. Particular emphasis was placed on role clarity, leadership, closed-loop communication, task delegation, and shared situational awareness, consistent with crisis resource management principles and team-based cardiac arrest care frameworks [34]. This alignment was intentional because the subsequent escape-room intervention was designed to build on the same competency structure through time-pressured, collaborative problem-solving tasks.

To preserve standardization, the workshop was delivered by the same instructional team using identical teaching materials, simulation equipment, and educational sequence for all participants. No group-specific adaptation was introduced at this stage. The workshop therefore served as a common preintervention platform for both groups before implementation of the trial-specific educational contrast. The workshop content was intentionally aligned with the competency domains later operationalized in the escape-room scenarios and TOSCE assessment framework, thereby creating continuity between foundational review, experiential intervention, and outcome measurement.

### CPR-EER intervention

One week after completion of the common CPR refresher workshop, participants allocated to the intervention arm received the CPR-EER training in the faculty’s clinical simulation laboratory. The intervention was designed as a gamified, experiential simulation grounded in team-based resuscitation principles, with the cognitive progression of tasks explicitly informed by Bloom’s revised taxonomy [35, 36]. Its overarching purpose was not only to rehearse psychomotor CPR skills, but also to strengthen role clarity, shared situational awareness, rapid decision-making, and team coordination during in-hospital cardiac arrest (IHCA) scenarios [37, 38].

The clinical simulation laboratory was reconfigured into a sequential escape-room environment consisting of three interconnected challenge stations. The thematic narrative placed participants in a high-stakes hospital emergency in which they were required to solve clinically relevant tasks within a limited time to unlock the final exit, thereby enhancing immersion and engagement while maintaining alignment with predefined educational objectives. Before entering the environment, all participants received a standardized pre-briefing in an adjacent room. This briefing introduced the operating rules, the 18-minute active time limit, the scoring procedure, the use of optional hints, expectations for team communication, and the requirement that each scenario be completed successfully before progression to the next stage. To preserve intervention fidelity, the pre-briefing script was delivered verbatim for all teams. The full pre-briefing script, operational rules, scenario details, and progression logic are provided in Supplementary File 1.

The intervention was delivered in preassigned, fixed teams. Team composition was determined before the session to promote balanced participation and to reflect the operational structure of a clinical resuscitation team. Within each team, one student was designated as the Team Leader on the basis of baseline team performance in the TOSCE, while the remaining members were assigned the standardized operational roles of Coordinator, Nurse A&B, Nurse C, Nurse D, and Nurse E, as specified in Supplementary File 1. These roles remained fixed throughout the intervention to promote role accountability, leadership development, and coordinated task execution. The intervention consisted of three sequential scenarios, each requiring teams to apply CPR knowledge and teamwork behaviors under time pressure. The scenarios were designed to promote progressive cognitive engagement, beginning with role recognition, continuing with emergency-response sequencing, and culminating in the identification and evaluation of errors within an advanced cardiac life support context. The pedagogical alignment of each scenario with its corresponding learning objectives, puzzle structure, and progression logic is summarized in Table 2.

**Table 2:** Pedagogical mapping of the CPR escape-room scenarios, learning objectives, puzzle mechanics, and progression logic

Scenario & Cognitive Level	Learning Objectives	Clinical / Gamified Task	Puzzle Mechanism & Codes	Transition / Exit Trigger
Scenario 1: Role Mapping Challenge (Bloom’s Level: Remember & Understand)	• Identify predefined roles in a resuscitation team. • Understand physical positions and core responsibilities around the bed.	• Match six role cards (Team Leader, Coordinator, Nurse A&B, Nurse C, Nurse D, and Nurse E) to their correct positions and responsibilities.	• Correct alignment reveals numeric codes on the reverse side of the cards. • The digits are entered in left-to-right sequence into Laptop 1. • A correct entry reveals the password for Laptop 2. • Hint system: yellow envelopes (5-point deduction per use).	• Entering the password in Laptop 2 unlocks access to Scenario 2.
Scenario 2: Emergency Response Simulation (Bloom’s Level: Apply)	• Execute initial resuscitation steps in the correct sequence. • Apply clinical guidelines to a patient-collapse scenario.	• Respond to a post-surgical patient found unresponsive. • Perform CPR-related actions in sequence, including assessment, code activation, patient positioning, backboard placement, and crash-cart preparation.	• After opening Laptop 2, participants proceed to the simulation task. • Numeric clues are hidden in physical locations in the room. • The compiled sequence is entered into Laptop 3. • A correct entry reveals the password for Laptop 4. • Hint system: red envelopes (5-point deduction per use).	• Entering the password in Laptop 4 unlocks access to Scenario 3.
Scenario 3: Error Identification & Evaluation (Bloom’s Level: Analyze & Evaluate)	• Analyze clinical logs for resuscitation errors. • Evaluate interventions against AHA guidelines.	• Review a written resuscitation transcript containing deliberate clinical, pharmacological, and communication errors. • Collaboratively identify and count the total number of errors.	• After opening Laptop 4, participants review the error-identification task. • The total number of correctly identified errors forms the master code, which is entered into Laptop 5. • Hint system: green envelopes (5-point deduction per use).	• A correct entry in Laptop 5 reveals the physical location of the mechanical key used to unlock the exit door.

**Fig. 2:**
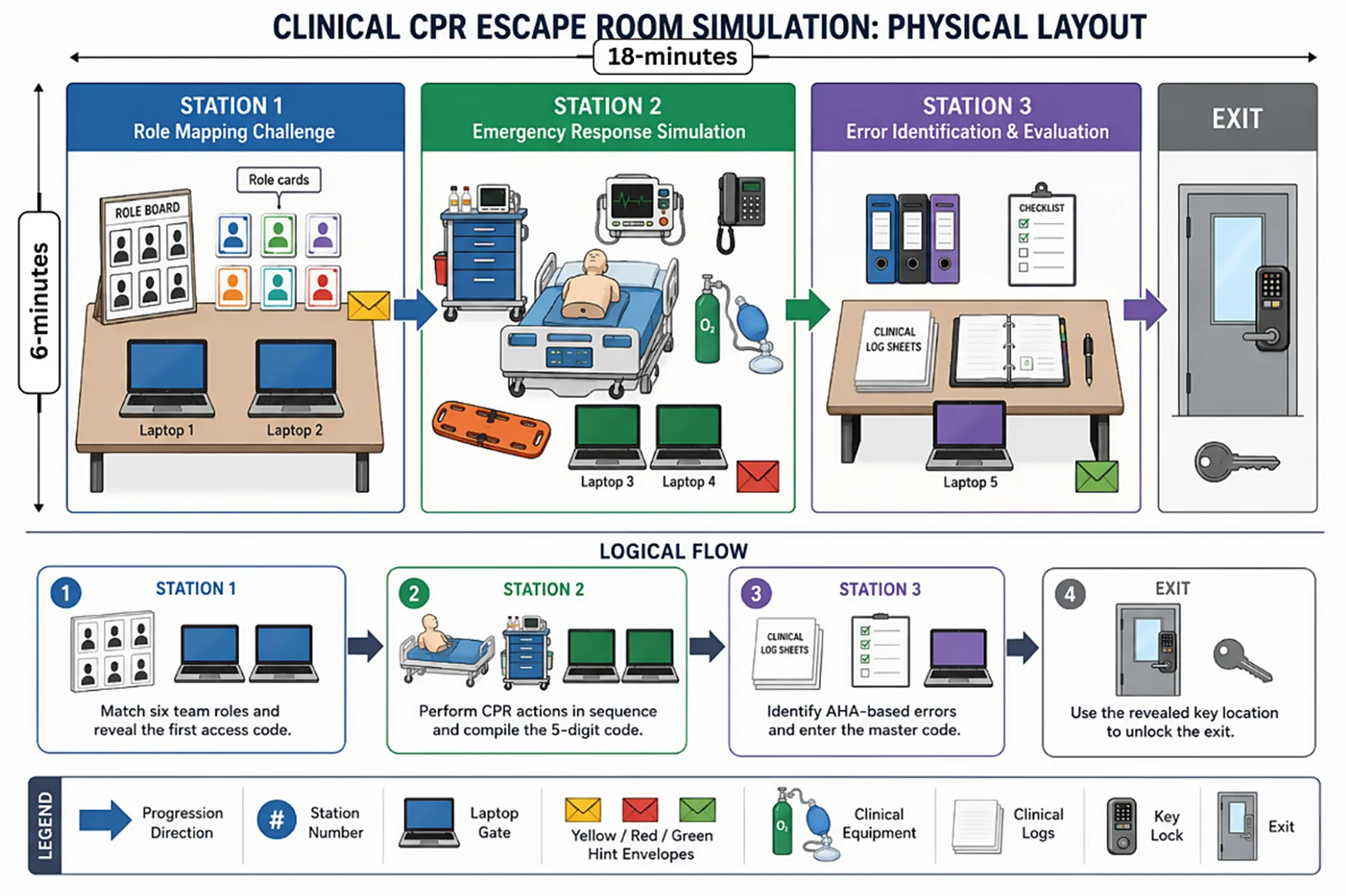
Physical layout and progression architecture of the CPR escape room simulation. The schematic depicts the three sequential stations, major clinical and task-related equipment, and the five network-disabled laptops used as progression gates. Participants first completed the role-mapping task and entered the resulting code into Laptop 1; the revealed password enabled access to Laptop 2 and the second station. In the emergency response station, the compiled action-sequence code was entered into Laptop 3, revealing the password for Laptop 4 and unlocking the third station. At the final station, the number of identified errors in the clinical log was entered into Laptop 5, revealing the location of the physical key used to unlock the exit door

Scenario progression was strictly linear and was controlled through an automated gatekeeping system comprising five network-disabled laptops. To ensure standardized navigation, participants used Laptops 1, 3, and 5 for the primary clinical code entries, whereas Laptops 2 and 4 functioned as intermediary password-protected access gateways that unlocked the subsequent stations after successful completion of the preceding task. This cryptographic progression logic ensured that teams could not advance until the specific clinical objectives of each station had been achieved. The physical and logical layout of the three sequential challenge stations, including the placement of all five laptops, the resuscitation mannequin, and the clue envelopes, is illustrated in Fig. 2.

To minimize facilitation bias and ensure inter-session consistency, a single trained faculty member served as the facilitator in a strictly observational capacity. The facilitator was not permitted to provide clinical guidance or unsolicited prompts. Their role was limited to delivering the pre-briefing, monitoring the 18-minute countdown, and administering the standardized color-coded hint system only when formally requested by the Team Leader. Each hint request resulted in a prespecified 5-point deduction from the team’s initial score of 100 points. To maintain environmental equivalence across team rotations, a standardized Environmental Reset Checklist was completed between sessions to ensure that each team encountered an identical physical baseline (Supplementary File 1, Sect. “7”). At the conclusion of the activity, each team participated in a structured debriefing based on the GAS (Gather, Analyze, Summarize) framework to consolidate learning, clarify errors, and link the experience to accepted CPR team-performance principles. Detailed descriptions of the scenario scripts, hint contents, and the CPR-EER operational scoring rubric are presented in Supplementary File 1.

### Intervention dose and delivery characteristics

The CPR-EER intervention was delivered as a single, highly structured educational session for each team. During this session, each team completed one full escape-room sequence comprising the three interlinked scenarios. Total active engagement time within the room was strictly limited to 18 min and did not include the standardized pre-briefing or the post-intervention GAS debriefing. To ensure consistency of delivery, all teams underwent the intervention under the same environmental setup, used identical resuscitation and monitoring equipment, followed the same progression order, received identical initial instructions, and were subject to the same timing rules, hint procedures, and facilitator protocols. The intervention was implemented by the same research team under uniform simulation conditions for all participants to enhance reproducibility and reduce implementation bias. Additional operational details related to scenario flow, room setup, pre-briefing, task structure, progression logic, and scoring are provided in Supplementary File 1. No protocol deviations occurred during the trial implementation; all teams completed the intervention as per the predefined sequence and timing.

### Delayed-intervention control group and fidelity procedures

Participants allocated to the control group did not receive the escape-room intervention during the primary comparison phase of the trial; instead, they continued with the routine undergraduate nursing curriculum and completed the same baseline and postintervention assessments as the intervention group over the identical study period. To address ethical and educational fairness considerations, the control group was scheduled to receive the same escape-room training experience immediately after postintervention data collection; accordingly, the comparator was defined as a delayed-intervention control. To preserve study fidelity and minimize cross-group contamination, several methodological safeguards were implemented. Group allocation was disclosed only after completion of the baseline knowledge and TOSCE assessments, thereby maintaining allocation concealment during the initial assessment phase. In addition, intervention sessions and control-group assessments were scheduled in a manner that minimized temporal overlap and reduced opportunities for interaction between participants in the simulation department. Participants in the intervention group were also formally instructed not to share details of the scenarios, codes, or puzzle mechanics with their peers, and access to the physical escape-room environment and related materials was strictly limited to scheduled sessions. Furthermore, both groups received the same 8-hour CPR refresher workshop before the intervention phase to ensure an equivalent educational baseline. Finally, outcome assessors remained masked to group allocation throughout the evaluation of all postintervention TOSCE assessments. The overall timeline of enrollment, allocation, interventions, and assessments for both groups is summarized in Table 1.

### Variables and outcomes

Study outcomes were assessed at baseline (T1) and one month after the intervention (T2). The primary outcome was team coordination in resuscitation, while secondary outcomes included role-specific performance, CPR knowledge, and participant satisfaction. The assessment framework was constructed on a single CPR competency blueprint shared across the common ALS workshop, the CPR-EER escape-room scenarios, the knowledge test, and the TOSCE stations. This blueprint covered recognition of cardiac arrest, role allocation, leadership, closed-loop communication, task delegation, adherence to ALS/CPR algorithms, technical execution of core resuscitation tasks, and post-resuscitation team management. Accordingly, the educational content, intervention tasks, and outcome measures were explicitly aligned with the same learning objectives and competency domains.

#### Primary outcome: team coordination in TOSCE

Team coordination during resuscitation was assessed using a standardized Team Objective Structured Clinical Examination (TOSCE) conducted in the simulation laboratory. The examination comprised three structured stations designed to evaluate complementary dimensions of team-based CPR: (1) allocation of resuscitation roles and bedside positioning (2), execution of CPR during a cardiac arrest scenario, and (3) post-resuscitation management and team communication. Participants performed in fixed six-member teams, with each member assuming one of six predefined roles: Team Leader, Coordinator, Nurse A&B, Nurse C, Nurse D, or Nurse E. To preserve comparability across assessments, team composition and role allocation were kept constant for each team across T1 and T2. Scoring emphasized role fidelity, communication clarity, task distribution, leadership behaviors, and collaborative decision-making [6, 39]. The examiner team consisted of six trained educators with expertise in emergency nursing and EMS training. Before data collection, all examiners completed a standardized calibration session using the same scoring manual, station scripts, and benchmark performances to promote scoring consistency. At each station, two examiners independently scored team performance while remaining masked to group allocation. Pre- and post-intervention assessments used equivalent station blueprints with identical competency targets, scoring structure, task sequence, time allocation, and difficulty level to minimize testing bias while avoiding simple recall from repeated exposure. Team coordination was scored using structured checklists developed in accordance with current advanced life support recommendations [8, 40]. The maximum total score across the three stations was 39. Discrepancies between paired examiner ratings were reviewed against the scoring criteria and resolved through consensus immediately after the station; where needed, a senior assessor adjudicated unresolved differences. The checklists underwent face and content validity review by a panel of ten experts, achieving item-level content validity index (I-CVI) scores ≥ 0.80 and Cronbach’s alpha ≥ 0.79. The complete TOSCE scenarios, parallel station blueprints, checklists, and examiner instructions are provided in Supplementary File 2.

#### Secondary outcomes: role-specific performance, knowledge, and satisfaction

- **Role-Specific Performance**: This was assessed concurrently within the TOSCE using checklists aligned with the specific responsibilities of each team member (e.g., airway management, defibrillation). This measure focused on individual enactment within the team context [30, 41]. The maximum score was 12, and the tool demonstrated satisfactory validity (I-CVI ≥ 0.80) and reliability (Cronbach’s alpha ≥ 0.79). Detailed checklists are in Supplementary File 2.
- **CPR Knowledge**: Measured using a 20-item multiple-choice questionnaire based on contemporary ALS guidelines [21–23]. Item construction was informed by Bloom’s cognitive taxonomy, targeting recall (20%), comprehension/application (65%), and higher-order analysis (15%). An appropriate blueprint was derived from the same CPR competency framework used for the ALS workshop, the escape-room scenarios, and the TOSCE, thereby ensuring alignment between instructional content and outcome measurement. The maximum score was 20. The tool achieved an I-CVI of 0.80–1.00 and a Cronbach’s alpha of 0.85.
- **Participant Satisfaction**: Measured in the intervention group using a 16-item validated questionnaire [42]. Scores ranged from 16 to 48, categorized as low (< 22), moderate [22–36], or high (> 37).

### Data collection procedure and statistical analysis

Data collection was performed at two time points: baseline (T1) and one month post-intervention (T2), as summarized in Table 1. At baseline, demographic characteristics, pre-intervention knowledge, and baseline TOSCE scores were recorded. Following the intervention phase, both groups underwent the same post-intervention assessments. In keeping with the randomized parallel-group design, the principal aim of the analysis was to estimate the between-group effect of the intervention on post-intervention outcomes while accounting for baseline values.

#### Analytic strategy and per-protocol framework

Statistical analyses were performed using SPSS version 26.0. The primary outcome, team coordination, was generated within intact six-member teams; therefore, the team was treated as the unit of analysis for the primary endpoint. Because meaningful assessment of team coordination was not possible when attendance was incomplete or team composition was disrupted, the primary analysis was conducted on a per-protocol basis using teams with complete evaluable outcome data. Participants or teams that did not complete the assigned educational pathway or the required outcome assessments were excluded from the primary analysis set, and all withdrawals and post-randomization exclusions were documented with reasons in the participant flow diagram. The intention-to-treat principle was considered during analysis planning; however, a full intention-to-treat analysis was not feasible for the primary endpoint because valid reconstruction of team-coordination scores was not possible for incomplete teams. Accordingly, missing data for the primary endpoint were not imputed. For secondary outcomes, analyses were based on available complete data for each prespecified measure. The trial prespecified one confirmatory primary outcome and several supportive secondary outcomes. Station-level scores were incorporated into the overall TOSCE framework defined a priori rather than analyzed as separate primary endpoints.

#### Statistical methods and rationale

Descriptive statistics were used to summarize study variables and participant characteristics, including means and standard deviations for approximately normally distributed variables, medians and interquartile ranges for skewed variables, and frequencies and percentages for categorical variables. Baseline characteristics were reported descriptively and were not subjected to significance testing. For the primary outcome, the between-group intervention effect on post-intervention team coordination was estimated using analysis of covariance (ANCOVA), with the post-intervention team coordination score entered as the dependent variable, study group as the fixed factor, and the corresponding baseline score as a covariate. This baseline-adjusted between-group comparison was prespecified as the primary inferential analysis and was selected in preference to separate within-group change tests. Secondary continuous outcomes were analyzed using the same baseline-adjusted framework whenever model assumptions were reasonably met, and adjusted mean differences with 95% confidence intervals were reported.

Because the primary endpoint was derived from structured team-based assessment across multiple stations and independent masked examiners, the prespecified total TOSCE score was used as the principal outcome metric. Station scores and examiner ratings were incorporated into this aggregate scoring structure according to the standardized rubric described in Supplementary File 2 and were not treated as separate confirmatory comparisons. This approach was adopted to preserve alignment with the competency-based assessment framework and to limit multiplicity from repeated testing across stations. Formal hypothesis testing was focused on the primary endpoint; analyses of secondary outcomes were interpreted as supportive. In addition, the individualized class average normalized gain (CANG) was calculated as an educational descriptive index using the formula: g = (posttest score - pretest score) / (maximum possible score - pretest score). CANG was treated as a supplementary measure and not as the primary estimator of the intervention effect.

#### Analytical considerations

A mixed-effects framework was considered during analysis planning because of the repeated-measure structure of the study. However, given the modest sample size, the team-based nature of the primary endpoint, and the use of an aggregated TOSCE score across stations and assessors, ANCOVA was selected as the main analytical approach because it provided a parsimonious and interpretable estimate of the between-group post-intervention effect while adjusting for baseline. The statistician worked with de-identified data and coded group labels, and group identity was not revealed until the primary analysis specification had been finalized. All tests were two-sided, and a p-value of < 0.05 was considered statistically significant [43, 44].

## Discussion

This protocol addresses an important gap in health professions education by developing and evaluating an educational escape room (EER) as a structured, team-based approach to CPR training for undergraduate nursing students. The study is grounded in the premise that effective resuscitation requires more than technical knowledge alone; it also depends on rapid role allocation, communication, shared situational awareness, and coordinated action under pressure. In this regard, the present intervention is designed not merely as an engaging educational activity, but as a deliberately structured learning experience intended to align game mechanics with clearly defined clinical learning outcomes. The use of Bloom’s taxonomy as an instructional framework supports this alignment by ensuring that the three sequential puzzles progressively engage learners at different cognitive levels, from recall and comprehension to application, analysis, and collaborative problem solving [45–47]. Such pedagogical structuring is important because the educational value of an EER depends on more than novelty or enjoyment; it depends on the coherence between the game design and the intended competencies.

Another distinctive feature of this protocol is the explicit integration of team management of CPR into both the intervention and the evaluation strategy. The escape-room scenarios have been designed to reinforce role clarity, communication, and coordinated task execution, while the TOSCE has been selected to assess these same competencies in a structured and observable manner [48, 49]. This conceptual alignment between intervention and outcome assessment strengthens the methodological coherence of the study and may enhance the interpretability of its findings. In addition, the use of both team-level and role-specific checklists, developed through psychometric procedures and presented in the supplementary materials, may provide a useful template for future researchers who aim to evaluate complex performance-based outcomes in simulation-based education. The broader literature on escape-room learning in health professions education suggests that this format is increasingly being used to support active participation, teamwork, and knowledge integration, although methodological heterogeneity remains substantial [50, 51]. Accordingly, this protocol contributes not only to CPR education, but also to the methodological literature on how gamified simulation should be designed and evaluated in rigorous educational research.

This study is also important because it focuses on CPR, a high-stakes clinical domain in which both individual competence and team performance are essential. While escape rooms have been used in various educational contexts, fewer studies have focused specifically on resuscitation training and even fewer have embedded teamwork as a primary educational target. The CPR-EER described in this protocol was therefore designed to provide a realistic, collaborative, and time-sensitive environment in which students must apply knowledge, interpret cues, distribute roles, and coordinate actions in a manner that reflects the demands of emergency care. Prior work on escape-room-based education in nursing has emphasized the importance of well-structured facilitation, clear instructional objectives, and careful alignment between puzzles and learning outcomes [52, 53]. The present protocol builds on that literature by placing team coordination at the center of both learning design and assessment, thereby extending the educational logic of EERs from general engagement toward clinically meaningful team performance.

From a curricular perspective, this protocol may also be valuable because it incorporates a delayed-intervention control condition and standardized baseline CPR refresher training for both groups [54, 55]. This design improves ethical acceptability while helping to ensure that observed differences are attributable to the added value of the escape-room format rather than to unequal exposure to core CPR content [56, 57]. In addition, the use of masked outcome assessment, standardized checklists, and a structured debriefing process enhances the internal consistency of the study and supports a more defensible interpretation of the eventual findings. The protocol may therefore offer a reproducible model for integrating game-based learning into emergency nursing education without sacrificing methodological rigor. Overall, this protocol was developed to generate evidence on whether a team-based CPR-EER could serve as a feasible, acceptable, and pedagogically coherent educational strategy for undergraduate nursing students. Beyond its immediate application to CPR, the study may also inform the broader development of simulation-based games for other high-acuity clinical situations in which communication, prioritization, and team coordination are critical.

### Strengths and limitations

A major strength of this protocol is its randomized controlled design with a delayed-intervention comparator, which supports internal validity while remaining ethically acceptable in an educational setting. Another strength is the use of an on-site, face-to-face learning environment, which is likely to provide stronger immersion and team interaction than purely virtual or remote formats. The study is also strengthened by the fact that both groups receive the same CPR refresher workshop before the intervention phase, which helps standardize baseline exposure and makes the incremental educational value of the escape-room intervention easier to interpret. In addition, the use of structured TOSCE stations, psychometrically developed checklists, and assessor masking helped reduce measurement bias and improve the reliability of outcome assessment. The availability of detailed intervention procedures and assessment tools in the supplementary files further enhances transparency and reproducibility.

At the same time, several limitations should be acknowledged. First, EERs are relatively resource-intensive and may require considerable time, space, and personnel for preparation, implementation, and facilitation [52]. This may limit scalability in settings with fewer simulation resources or larger student cohorts. Second, complete participant blinding was not feasible in this type of intervention, and although several steps were taken to minimize contamination, some exchange of information between groups could not be fully prevented. Accordingly, the study should be described as assessor-blinded rather than double-blinded. Third, the intervention is specifically focused on CPR team management, so the findings may not be directly generalizable to other emergency or critical care scenarios. Although the educational structure may be adaptable to other clinical topics, this remains to be tested in future research.

A further limitation is that the protocol emphasizes short-term educational outcomes, and the extent to which the intervention supports long-term retention or transfer to real clinical performance remains uncertain. For that reason, future studies should examine whether the benefits of the CPR-EER persist over time and whether similar escape-room structures can be adapted for other high-stakes clinical competencies.

## Supplementary Information

Below is the link to the electronic supplementary material.

Supplementary Material 1

Supplementary Material 2

## Data Availability

No datasets were generated or analysed during the current study.

## Abbreviations

ALS: Advanced Life Support
BLS: Basic Life Support
CPR: Cardiopulmonary Resuscitation
CPR: -EER Cardiopulmonary Resuscitation-Educational Escape Room
EER: Educational Escape Room
I-CVI: Content Validity Index of each Item
TOSCE: Team structured objective clinical examination
SPIRIT: Standard Protocol Items for Randomized Trials
