# Supplementary material for: Design and protocol of a randomized assessor-blinded controlled trial evaluating an educational escape room for individual and team cardiopulmonary resuscitation skills among nursing students
Source: BMC Nurs. 2026 Jul 29;25:1021. doi: 10.1186/s12912-026-05151-4 (PMC13711775; doi:10.1186/s12912-026-05151-4)
Supplement: Supplementary file 1 [file 12912_2026_5151_MOESM1_ESM.docx]

**Supplementary File 1: Clinical Scenario Specification, Task Logic, and Evaluation Rubrics for the CPR-EER Protocol**

**Section 1: Pre-Briefing Script & Environmental Setup**

**1.1 Standardized Pre-Briefing Script**

*Delivered verbatim by the facilitator to all teams in the holding room 10 minutes prior to entry.*

"Welcome to the CPR Escape Room Simulation. Before you begin, please listen carefully to the operating parameters of this activity:

1. **Team Structure:** You have been assigned specific clinical roles based on your baseline TOSCE assessments. You must remain in these roles throughout the scenarios. The designated Team Leader is responsible for coordinating the team’s response.
2. **Time Limit:** You have exactly 18 minutes to complete all three sequential scenarios and escape the room. Progression is strictly linear; you cannot attempt a scenario until the previous one is successfully unlocked via the designated laptops.
3. **Hints System:** If your team becomes stuck, you may open the color-coded hint envelopes located at each station (Yellow for Station 1, Red for Station 2, Green for Station 3). Opening a hint envelope requires you to solve a quick clinical question written on the outside. If you answer correctly, you may open the envelope for a hint, incurring a 5-point deduction from your starting score of 100 points. If your answer is incorrect, you will not receive the hint, but the 5-point deduction will still apply.
4. **Equipment Handling:** Treat all simulation equipment, including the high-fidelity Ambu Man mannequin and clinical devices, with professional care. No physical force is required to open any lockboxes or doors; all locks are opened via numeric codes or keys retrieved through clinical problem-solving.

Your countdown will begin the moment you cross the threshold of the simulation lab. Your mission is detailed in the briefing note on the entry desk. Good luck."

**1.2 Environmental & Equipment Setup**

- **Simulation Room Dimensions:** 6 m×5 m configured as a standard surgical ward room.
- **Mannequin:** Ambu Man advanced resuscitation training mannequin positioned on a standard hospital bed.
- **Resuscitation Equipment:** Crash cart (equipped with a functional manual defibrillator/monitor, airway adjuncts, bag-valve-mask, IV access supplies, and empty pre-labeled medication vials), hard backboard (cardiac massage board), wall-mounted telephone.
- **Gatekeeping Interfaces:** 5 network-disabled laptops serving as sequential physical and logical progression gates (Laptops 1 through 5).

**Section 2: Clinical Scenario 1 – Role Mapping Challenge (Remember & Understand)**

**2.1 Scenario Description & Objective**

The objective of this scenario is to assess the team’s ability to recall and comprehend the predefined roles, responsibilities, and physical positioning of members within a resuscitation team according to the intervention design and accepted CPR team-performance principles.

**2.2 Physical Execution Mechanics**

Participants face a table featuring a transparent board displaying six role cards: **Team Leader, Coordinator, Nurse A&B, Nurse C, Nurse D, and Nurse E**. Teams must correctly match each role card with two corresponding sets of cards placed on the table: one describing the principal responsibility of that role, and the other indicating the correct physical position of that team member around the patient during CPR.

Once all cards are correctly aligned, the board is flipped, revealing a numeric password on the back of each card. When read from left to right, this sequence forms the code to unlock Scenario 2. Any incorrect alignment disrupts the password sequence and prevents progression. Teams may request a hint by opening the yellow envelope assigned to Station 1. Each hint request incurs a standardized score deduction, and access to the clue is granted only after attempting the clinical question printed on the envelope exterior.

**Table S1: Role Mapping Cards and Key Grid Coordinates**

| Role Title | Responsibilities Statement | Grid Code (Position) | Digit |
| --- | --- | --- | --- |
| Team Leader | Assigns roles, oversees team performance, and makes treatment decisions. | Position 1 | **4** |
| Coordinator | Maintains overall coordination, supports communication, and ensures task integration. | Position 2 | **8** |
| Nurse A&B | Performs high-quality chest compressions and rotates roles to prevent fatigue. | Position 3 | **2** |
| Nurse C | Manages the airway and delivers ventilations using bag-valve-mask (BVM) support. | Position 4 | **9** |
| Nurse D | Establishes vascular access and prepares/administers medications. | Position 5 | **5** |
| Nurse E | Operates the monitor/defibrillator and records key event timings. | Position 6 | **7** |

**2.3 Transition Code**

The resulting code sequence entered into **Laptop 1** is: **4-8-2-9-5-7**.

- **System Action:** Upon entering this code, Laptop 1 displays: *“Access Granted. Laptop 2 Password is: ALIVE2026”*.
- **Progression:** The team enters this password into **Laptop 2** (the entry gateway to Station 2) to physically proceed to the next stage.

**Section 3: Clinical Scenario 2 – Emergency Response Simulation (Apply)**

**3.1 Scenario Case Presentation**

Mr. X, a 75-year-old male, Post-Op Day 3, collapsed after attempting to stand. The team must execute the early access and high-quality CPR steps in correct sequence.

**3.2 Clinical Action Sequence & Code Discovery**

To find the digits, the team must perform the following actions. The digits are physically hidden at the location of each correct action:

1. **Step 1 (Check Consciousness & Pulse):** Digit **3** (found under the mannequin’s collar).
2. **Step 2 (Activate Code Blue):** Digit **1** (found under the wall-mounted telephone handset).
3. **Step 3 (Transfer Patient to Bed):** Digit **6** (found on the mannequin’s left shoulder).
4. **Step 4 (Position Crash Cart):** Digit **8** (found on the back panel of the crash cart).
5. **Step 5 (Place Cardiac Board):** Digit **2** (engraved on the hard backboard/cardiac massage board).

**3.3 Transition Code**

The compiled code sequence entered into **Laptop 3** is: **3-1-6-8-2**.

- **System Action:** Upon entering this code, Laptop 3 displays: *“Access Granted. Laptop 4 Password is: ACUTECARE2026”*.
- **Progression:** The team enters this password into **Laptop 4** (the entry gateway to Station 3) to unlock the clinical resuscitation log.

**Section 4: Clinical Scenario 3 – Error Identification (Analyze & Evaluate)**

**4.1 Scenario Description**

Teams review a comprehensive 48-step clinical log (Table S2) to identify all technical and non-technical deviations from AHA ACLS guidelines. The total count of identified errors serves as the entry code for the final laptop.

**Table S2: Complete Resuscitation Log and Error Analysis**

| Step | Documented Action | Status | Guideline Deviation / Rationale |
| --- | --- | --- | --- |
| 1 | Assessed responsiveness and breathing simultaneously | Correct | Complies with standard AHA assessment. |
| 2 | Performed carotid pulse check for 15 seconds | **ERROR** | Pulse check must not exceed 10 seconds. |
| 3 | Called for defibrillator and emergency cart | Correct | Appropriate early activation of resources. |
| 4 | Positioned backboard under patient’s thorax | Correct | Essential for high-quality chest compressions. |
| 5 | Initiated chest compressions at a rate of 90/min | **ERROR** | Comp. rate must be between 100 and 120/min. |
| 6 | Delivered compressions at a depth of 5.5 cm | Correct | Meets the 5–6 cm depth requirement. |
| 7 | Delivered ventilations with each breath lasting 3 seconds | **ERROR** | Breath delivery must take approximately 1 second. |
| 8 | Maintained a compression-to-ventilation ratio of 30:2 | Correct | Standard ratio for advanced airway-free CPR. |
| 9 | Checked rhythm after 2 minutes of CPR | Correct | Standard cycle interval. |
| 10 | Adjusted oxygen flow rate to 5 L/min | **ERROR** | Bag-valve-mask requires high-flow O2O_2O2 (10–15 L/min). |
| 11 | Applied defibrillator self-adhesive pads | Correct | Recommended for monitoring and rapid shock delivery. |
| 12 | Identified Ventricular Fibrillation (VF) rhythm | Correct | Correct identification of a shockable rhythm. |
| 13 | Applied pediatric pads to the adult mannequin | **ERROR** | Adult pads are required for patients >8>8>8 years old. |
| 14 | Charged defibrillator to 200 Joules (Biphasic) | Correct | Standard initial biphasic energy level. |
| 15 | Delivered shock while team member held the IV line | **ERROR** | Bed must be completely clear of all personnel during shock. |
| 16 | Resumed chest compressions immediately after shock | Correct | CPR must be resumed immediately without rhythm recheck. |
| 17 | Established peripheral IV access | Correct | Standard route for drug administration. |
| 18 | Identified persistent VF rhythm after 2 minutes | Correct | Standard evaluation point. |
| 19 | Administered Epinephrine 1mg IV after the first shock | **ERROR** | Epinephrine is administered after the second shock in VF. |
| 20 | Flushed IV line with 20 mL of normal saline | Correct | Necessary to ensure drug delivery into central circulation. |
| 21 | Resumed CPR for another 2-minute cycle | Correct | Maintenance of resuscitation cycles. |
| 22 | Charged defibrillator to 360 Joules on a Biphasic device | **ERROR** | Biphasic maximum is typically 200J; 360J is for monophasic. |
| 23 | Delivered second shock successfully | Correct | Appropriate shockable rhythm protocol. |
| 24 | Administered Amiodarone 300mg IV bolus | Correct | Correct first dose for refractory VF. |
| 25 | Inserted supraglottic airway device (LMA) | Correct | Advanced airway management. |
| 26 | Provided continuous compressions without pauses | Correct | Standard with advanced airway in place. |
| 27 | Delivered 1 breath every 6 seconds (10 breaths/min) | Correct | Correct ventilation rate for advanced airway. |
| 28 | Administered Amiodarone as a slow IV drip over 15 mins | **ERROR** | Amiodarone in cardiac arrest must be a rapid IV bolus. |
| 29 | Re-evaluated rhythm after 2 minutes | Correct | Standard cycle check. |
| 30 | Identified rhythm change to Asystole | Correct | Correct identification of non-shockable rhythm. |
| 31 | Charged defibrillator to 200 Joules | Correct | Preparation for potential shock (if rhythm changes). |
| 32 | Delivered a shock to the Asystole rhythm | **ERROR** | Asystole is a non-shockable rhythm; do not shock. |
| 33 | Resumed CPR immediately after the rhythm check | Correct | Prompt resumption of cycle. |
| 34 | Administered Epinephrine 1mg IV | Correct | Epinephrine is indicated for non-shockable rhythms. |
| 35 | Checked ETCO2 reading (measured at 18 mmHg) | Correct | Good indicator of CPR quality and perfusion. |
| 36 | Confirmed tube placement via bilateral breath sounds | Correct | Crucial step for advanced airway validation. |
| 37 | Maintained chest compression fraction at 85% | Correct | Meets target threshold (>60%>60\%>60%, ideally >80%>80\%>80%). |
| 38 | Paused chest compressions for 15 seconds to insert LMA | **ERROR** | Compressions should not be paused >10>10>10 seconds for airway. |
| 39 | Conducted team rotation of compressor role | Correct | Compressor changed every 2 minutes. |
| 40 | Checked rhythm after 2 minutes | Correct | Standard interval. |
| 41 | Identified Sinus Rhythm on the monitor | Correct | Detection of return of spontaneous circulation (ROSC). |
| 42 | Performed pulse check during active chest compressions | **ERROR** | Pulse check during CPR leads to false positive pulses. |
| 43 | Paused CPR to verify carotid pulse | Correct | Correct action once a organized rhythm is identified. |
| 44 | Verified pulse present, BP=95/60 mmHg | Correct | Confirmation of ROSC. |
| 45 | Resumed chest compressions immediately after ROSC | **ERROR** | Compress. must stop; transition to post-ROSC care. |
| 46 | Initiated targeted temperature management protocols | Correct | Standard post-ROSC neuroprotective therapy. |
| 47 | Secured chest X-ray to confirm advanced airway position | Correct | Essential post-resuscitation placement confirmation. |
| 48 | Documented resuscitation event details | Correct | Final required step. |

**4.2 Transition Code**

- **Total Identified Errors:** **13**
- **System Action:** Entering **13** into **Laptop 5** unlocks the terminal interface, revealing the exact coordinates of the physical exit key.

**Section 5: Performance Evaluation Rubric (100-Point Scale)**

**Table S3: Scoring Rubric**

| Assessment Dimension | Metric / Criteria | Max Points |
| --- | --- | --- |
| 1. Scenario Accuracy | Successful completion of all 3 sequential scenarios. | 45 |
| 2. Timeliness | Completion within the 18-minute limit (Bonus/Penalty: +1 pt/min remaining; −1 pt/min over). | ±6 |
| 3. Team Collaboration | Non-technical skills (Role clarity, closed-loop communication, mutual support). | 15 |
| 4. Adherence | Clinical safety and guideline-compliant action sequencing. | 15 |
| 5. Hint Penalties | Standardized deduction per hint used (−5-5−5 points per hint, up to 9 hints maximum). | Up to -45 |
| Total Scale |  | **100** |

**Section 6: Standardized Environmental Reset Checklist**

*This protocol must be executed in full by the facilitator during the 15-minute transition window between team rotations to guarantee absolute environmental standardization.*

**Table-Top & Card Resets (Station 1)**

- Retrieve all six role-mapping cards from the transparent board or surrounding tables.
- Shuffle the role cards thoroughly and place them in a randomized stack beside the board.
- Ensure the board is wiped clean of any physical pencil/pen markings or alignment guides left by the previous team.

**Cryptographic & Device Resets (All Laptops)**

- **Laptop 1:** Reboot the program to the initial locked interface requiring the 6-digit sequence. Ensure prior inputs are cleared.
- **Laptop 2:** Log out of the interface, returning the screen to the primary password protection prompt.
- **Laptop 3:** Reset the software interface to the blank 5-digit entry field.
- **Laptop 4:** Close all active clinical log windows and return the screen to the initial password lock gate.
- **Laptop 5:** Clear the entry field, returning the screen to the final error-count prompt.

**Simulation & Environmental Material Resets (Station 2)**

- **Mannequin Position:** Return the high-fidelity Ambu Man to a flat, supine position in the center of the bed with the head flat. Disconnect any physical chest pads or electrodes applied.
- **Crash Cart:** Relocate the crash cart to its designated, marked baseline position on the left side of the room. Place all airway adjuncts, bag-valve-masks, and empty pre-labeled medication vials back into their exact designated drawers.
- **Cardiac Board:** Retrieve the hard backboard (cardiac massage board) and place it upright behind the patient bed, ensuring the engraved digit is not immediately visible without physical inspection.
- **Physical Clues:** Verify that the physical digits are securely positioned in their pre-determined hidden locations (i.e., collar of mannequin, under the phone handset, on the mannequin’s left shoulder, back panel of the cart, and on the cardiac board).

**Document & Hint Envelopes Resets (Station 3 & Exit)**

- **Clinical Logs:** Collect all clinical log files, transcript sheets, and scratch papers used by the previous team and replace them with fresh, unmarked clinical logs (Table S2).
- **Hint Envelopes:** Retrieve any opened hint envelopes (Yellow, Red, or Green). Replace them with newly sealed, identical envelopes containing the clinical prerequisite questions on the exterior and the physical hints on the interior.
- **Key & Door Lock:** Retrieve the mechanical key from Laptop 5’s revealed location. Lock the physical exit door from the inside and secure the mechanical key back into its standard concealed compartment.
