# Supplementary material for: Design and protocol of a randomized assessor-blinded controlled trial evaluating an educational escape room for individual and team cardiopulmonary resuscitation skills among nursing students
Source: BMC Nurs. 2026 Jul 29;25:1021. doi: 10.1186/s12912-026-05151-4 (PMC13711775; doi:10.1186/s12912-026-05151-4)
Supplement: Supplementary file 2 [file 12912_2026_5151_MOESM2_ESM.docx]

**Supplementary File 2**

**Team Objective Structured Clinical Examination (TOSCE): Station Scenarios, Required Equipment, Examiner Instructions, Expected Team Roles, and Scoring Checklists**

1. Purpose of This Supplementary File

This supplementary file provides the complete operational framework of the Team Objective Structured Clinical Examination (TOSCE) used in the present study to assess team-based cardiopulmonary resuscitation (CPR) performance among senior undergraduate nursing students. It is presented to enhance methodological transparency, support reproducibility, and enable readers to appraise the structure, implementation, and scoring logic of the assessment in sufficient detail for potential replication.

The TOSCE was designed to assess two interrelated domains: individual role-specific performance and overall team coordination during simulated resuscitation. To reflect the multidimensional and interdependent nature of CPR teamwork, the examination comprised three structured stations addressing: (1) role identification and bedside positioning, (2) resuscitation management during cardiac arrest, and (3) immediate post-resuscitation care.

This supplementary file includes the standardized station scenarios, required equipment, expected role responsibilities, examiner instructions, and team-level and individual-level scoring checklists used during assessment.

2. Relationship of the TOSCE to the Study Outcomes

In this trial, team coordination during resuscitation was the primary outcome and was assessed using the TOSCE at baseline and one month after the intervention. Participants completed the examination in fixed six-member teams, and each student performed a predefined role within the resuscitation team.

The TOSCE checklists were developed to assess observable behaviors relevant to coordinated resuscitation performance, including role identification, task execution, communication, timing, role fidelity, and integration of actions within the team process. Accordingly, this appendix functions as the operational assessment companion to the outcomes reported in the main article, particularly those related to role-specific performance and team coordination.

3. Standardization of the TOSCE

All three TOSCE stations were standardized with respect to scenario structure, required materials, role allocation, and scoring procedures. Each station included:

- a clearly defined station objective;
- a standardized student instruction or scenario prompt;
- a required equipment checklist;
- a team performance checklist;
- an overall team performance checklist, where applicable; and
- individual role-specific performance checklists.

This standardized structure was used to ensure consistency across teams and assessment sessions and to minimize variability unrelated to the intervention.

4. Team Structure and Assigned Roles

Participants completed the TOSCE in fixed six-member teams. Within each team, students were assigned predefined roles consistent with the team structure used in the study. These roles were:

- Team Leader
- Coordinator
- Nurse A&B
- Nurse C
- Nurse D
- Nurse E

Because the assessment was intended to evaluate performance within assigned team roles rather than isolated individual skills alone, particular emphasis was placed on role fidelity, coordinated action, communication, and integration of each member’s contribution within the team process.

5. General Instructions for Examiners

Performance at each station was assessed by trained examiners using predefined structured checklists. Examiners were instructed to base all ratings exclusively on directly observable performance during the simulation and to avoid inferences beyond what was demonstrated by the participant or team.

Examiners were instructed to:

- assess observable actions, communication behaviors, task completion, and adherence to assigned roles;
- score all items according to the predefined criteria for the relevant station;
- evaluate both technical performance and role-appropriate team functioning;
- avoid discussing scores with a co-examiner during active scoring; and
- complete all scoring forms independently.

As reported in the main article, six trained examiners participated in the assessment process, and all were blinded to group allocation. Two examiners independently completed the role-specific and team-coordination checklists at each station. Because the main article did not report a quantitative inter-rater reliability coefficient, this appendix retains the original scoring structure and assessment logic without introducing unsupported statistical claims.

6. Scoring Principles

The TOSCE used structured checklists to assess both team-level and role-specific performance. Depending on the nature of the item, three scoring formats were applied.

6.1. Five-Point Likert Scale

Most station items were scored using a 5-point Likert scale:

- 5 = Excellent
- 4 = Good
- 3 = Average
- 2 = Poor
- 1 = Very Poor

This scale was used for items assessing the quality, completeness, correctness, appropriateness, and timeliness of observed performance.

Suggested interpretation:

- 5 indicates correct, complete, timely, and well-coordinated performance;
- 4 indicates minor deficiencies that do not substantially impair task performance;
- 3 indicates partially correct, delayed, or inconsistently coordinated performance;
- 2 indicates markedly incomplete or weak performance; and
- 1 indicates omitted, incorrect, or unacceptable performance.

6.2. Three-Point Global Team Rating

Selected overall team-performance items were rated using a 3-point global scale:

- 5 = Acceptable
- 3 = Below expectations
- 1 = Unacceptable

This format was used for broader judgments regarding team coordination, role integration, and overall station performance.

6.3. Dichotomous Scoring

For selected discrete actions, particularly in Station 1, some items were scored dichotomously as:

- Yes
- No

This format was reserved for behaviors that could be clearly classified as performed or not performed.

7. Scope of Observation and Rating

The checklists in this supplementary file were designed to prioritize observable performance rather than presumed knowledge or generalized competence. Wherever possible, checklist items were framed as visible and assessable behaviors. Examiners were therefore expected to score what participants did, said, demonstrated, or completed during the station, rather than what they were presumed to know.

This principle is especially important in role-based simulation, where effective performance depends not only on technical actions but also on communication, timing, task coordination, and adherence to assigned responsibilities within the team.

8. Overview of the Three TOSCE Stations

The TOSCE consisted of the following three stations:

- Station 1: Identification of Resuscitation Team Roles and Bedside Positioning

Assesses the team’s ability to identify resuscitation roles, match role-specific duties, and determine correct bedside positioning.

- Station 2: Resuscitation of a Patient with Cardiac Arrest

Assesses coordinated management of an adult in-hospital cardiac arrest, including leadership, chest compressions, airway support, medication administration, defibrillation safety, documentation, and family support.

- Station 3: Post-Resuscitation Management

Assesses immediate management after return of spontaneous circulation (ROSC), including airway and ventilation support, hemodynamic and cardiac assessment, documentation, transfer coordination, temperature management as applicable, and family support.

9. Use of This Appendix for Reproducibility

This supplementary file is provided to enable readers, educators, and researchers to understand the content and structure of the TOSCE in sufficient detail for methodological appraisal and potential replication. The role-based assessment framework, standardized station format, equipment requirements, and scoring logic presented here reflect the examination model used in the study and are reported to strengthen reproducibility and implementation transparency.

Appendix Table A. Expected Duties by Team Role

| Role | Expected duties |
| --- | --- |
| Team Leader | Identifies the clinical situation; directs the resuscitation process; assigns or confirms roles; supervises adherence to the resuscitation sequence; monitors timing and task progression; ensures compressor rotation when indicated; instructs shock safety precautions before defibrillation; coordinates decision-making; and communicates with the family when appropriate. |
| Coordinator | Supports team organization and workflow; facilitates communication between ward personnel and the resuscitation team; documents key events and timing; assists with logistical arrangements; coordinates patient transfer when indicated; and helps maintain process continuity during and after resuscitation. |
| Nurse A&B | Assumes primary airway and ventilation responsibilities; positions the patient for airway management; maintains airway patency using appropriate adjuncts when indicated; applies the bag-mask device effectively; delivers ventilation at the appropriate rate; and supports oxygenation during resuscitation and post-resuscitation care. |
| Nurse C | Assumes primary circulation-related responsibilities; places the CPR backboard when required; performs chest compressions with appropriate technique; assesses cardiac rhythm when indicated; performs defibrillation when indicated and instructed; and supports circulation-focused actions during the resuscitation process. |
| Nurse D | Assumes intravenous access and medication-related responsibilities; obtains peripheral intravenous access; prepares required medications and related equipment; administers medications according to instruction and clinical indication; records administered drugs, doses, and timing; and assists with treatment-related measures during post-resuscitation care. |
| Nurse E | Provides supportive and auxiliary functions within the resuscitation team; assists with chest compressions or defibrillation when required; helps maintain workflow around the bedside; supports the patient’s family emotionally and practically when appropriate; facilitates respectful family communication or presence consistent with local policy; and contributes to dignified patient care. |

**Station 1. Identification of Resuscitation Team Roles and Bedside Positioning**

Station Objective

To assess the team’s ability to identify resuscitation roles, match role-specific duties, and determine the correct position of each member around the patient bedside.

Student Instruction

In this station, the duties of the resuscitation team members are presented on a board in a disorganized and non-classified format. First, identify your role within the resuscitation team. Then, select the duties related to your role from the list provided, write them on the back of the corresponding role card, and place the card around the mannequin according to the correct bedside position for that role.

1. Required Equipment Checklist

| No. | Required equipment | Available | Not available |
| --- | --- | --- | --- |
| 1 | Hospital bed |  |  |
| 2 | Patient mannequin for basic simulation; no special technical features required; positioned at the center of the bed |  |  |
| 3 | Board or poster containing the undifferentiated list of resuscitation team duties |  |  |
| 4 | A5 role cards with one role written on the front of each card: Team Leader, Coordinator, Nurse C, Nurse A&B, Nurse D, and Nurse E |  |  |
| 5 | Table |  |  |
| 6 | Pens (6) |  |  |

2. Team Performance Checklist

| No. | Assessment item | 5 Excellent | 4 Good | 3 Average | 2 Poor | 1 Very Poor |
| --- | --- | --- | --- | --- | --- | --- |
| 1 | The Team Leader correctly identified and recorded the duties assigned to this role |  |  |  |  |  |
| 2 | The Coordinator correctly identified and recorded the duties assigned to this role |  |  |  |  |  |
| 3 | Nurse C correctly identified and recorded the duties assigned to this role |  |  |  |  |  |
| 4 | Nurse A&B correctly identified and recorded the duties assigned to this role |  |  |  |  |  |
| 5 | Nurse D correctly identified and recorded the duties assigned to this role |  |  |  |  |  |
| 6 | Nurse E correctly identified and recorded the duties assigned to this role |  |  |  |  |  |
| 7 | The Team Leader correctly determined the bedside position for this role |  |  |  |  |  |
| 8 | The Coordinator correctly determined the bedside position for this role |  |  |  |  |  |
| 9 | Nurse C correctly determined the bedside position for this role |  |  |  |  |  |
| 10 | Nurse A&B correctly determined the bedside position for this role |  |  |  |  |  |
| 11 | Nurse D correctly determined the bedside position for this role |  |  |  |  |  |
| 12 | Nurse E correctly determined the bedside position for this role |  |  |  |  |  |

3. Overall Team Performance Checklist

| No. | Assessment item | 5 Acceptable | 3 Below expectations | 1 Unacceptable |
| --- | --- | --- | --- | --- |
| 1 | Team members identified their roles in a coordinated manner and within an appropriate time |  |  |  |
| 2 | Team members determined their bedside positions in a coordinated manner and in accordance with their assigned duties |  |  |  |

4. Individual Performance Checklist for Each Team Member

| No. | Assessment item | Yes | No |
| --- | --- | --- | --- |
| 1 | Accepted the assigned role based on team coordination and discussion |  |  |
| 2 | Correctly identified and recorded the duties corresponding to the assigned role |  |  |
| 3 | Correctly identified the bedside position corresponding to the assigned role |  |  |

**Station 2. Resuscitation of a Patient with Cardiac Arrest**

Station Objective

To assess team-based management of an adult in-hospital cardiac arrest, including leadership, chest compressions, airway support, defibrillation safety, medication administration, documentation, and family support.

Student Instruction

In this station, you are confronted with a scenario that requires you to act according to real clinical conditions and the assigned roles of the resuscitation team members.

You are the nurse responsible for a 52-year-old male patient with a history of cardiovascular disease and type 2 diabetes mellitus who has been admitted to the internal medicine ward for management of hyperglycemia. At 12:30 a.m., the patient’s family member calls you and reports nausea and vomiting. The patient appears pale and diaphoretic. While you are monitoring the patient and assessing vital signs, the patient develops a decreased level of consciousness and becomes unresponsive. You check the carotid pulse and detect no pulse. You ask the ward service staff member to activate the resuscitation code. The clinical supervisor and nursing staff arrive at the bedside according to the hospital code protocol. Due to another emergency, the emergency physician is temporarily unavailable. Demonstrate how you would manage this situation with the assistance of the resuscitation team.

1. Required Equipment Checklist

| No. | Required equipment | Available | Not available |
| --- | --- | --- | --- |
| 1 | Hospital bed |  |  |
| 2 | CPR mannequin; if available, a mannequin with performance feedback capability |  |  |
| 3 | Emergency trolley/crash cart fully stocked with required medications and equipment, positioned at a standard distance from the bed |  |  |
| 4 | Oxygen source (central or portable) |  |  |
| 5 | Patient chart/medical record |  |  |
| 6 | Pens (2) |  |  |
| 7 | Bedside table in front of the patient’s bed |  |  |
| 8 | Presence of a family member/relative as part of the simulation |  |  |

2. Team Performance Checklist

| No. | Assessment item | 5 Excellent | 4 Good | 3 Average | 2 Poor | 1 Very Poor |
| --- | --- | --- | --- | --- | --- | --- |
| 1 | A CPR backboard was correctly placed under the patient’s chest |  |  |  |  |  |
| 2 | The Team Leader clearly assigned team roles |  |  |  |  |  |
| 3 | Chest-compression timing was properly managed, including compressor rotation every 2 minutes |  |  |  |  |  |
| 4 | The team members responsible for ventilation and chest compressions coordinated effectively and counted aloud when appropriate |  |  |  |  |  |
| 5 | Medications were administered at the appropriate time according to the resuscitation process |  |  |  |  |  |
| 6 | A clear verbal safety warning was given before shock delivery |  |  |  |  |  |
| 7 | Team members performed actions consistent with their assigned roles and the resuscitation sequence |  |  |  |  |  |
| 8 | Equipment and personnel were positioned appropriately within the resuscitation area |  |  |  |  |  |
| 9 | One team member continuously documented major events, medications, and timing during resuscitation |  |  |  |  |  |
| 10 | One team member was assigned to provide support to the patient’s family member |  |  |  |  |  |

3. Overall Team Performance Checklist

| No. | Assessment item | 5 Acceptable | 3 Below expectations | 1 Unacceptable |
| --- | --- | --- | --- | --- |
| 1 | Coordination among team members was maintained throughout the resuscitation process |  |  |  |
| 2 | The performance of each team member was consistent with the assigned role in the resuscitation team |  |  |  |

4. Individual Performance Checklist: Team Leader

| No. | Assessment item | 5 Excellent | 4 Good | 3 Average | 2 Poor | 1 Very Poor |
| --- | --- | --- | --- | --- | --- | --- |
| 1 | Clearly assigned roles to team members |  |  |  |  |  |
| 2 | Positioned appropriately in relation to the patient and the team |  |  |  |  |  |
| 3 | Directed team actions in accordance with the duties assigned to each role |  |  |  |  |  |
| 4 | Effectively managed the resuscitation process according to the patient’s condition |  |  |  |  |  |
| 5 | Ensured rotation of the chest compressor every 2 minutes to maintain compression quality |  |  |  |  |  |
| 6 | Identified the appropriate team member to perform defibrillation when indicated |  |  |  |  |  |
| 7 | Instructed oxygen clearance and team safety precautions before shock delivery |  |  |  |  |  |
| 8 | Communicated with the patient’s family member regarding events and the patient’s condition when appropriate |  |  |  |  |  |

5. Individual Performance Checklist: Nurse A&B

| No. | Assessment item | 5 Excellent | 4 Good | 3 Average | 2 Poor | 1 Very Poor |
| --- | --- | --- | --- | --- | --- | --- |
| 1 | Positioned appropriately around the patient’s bed |  |  |  |  |  |
| 2 | Positioned the patient appropriately for airway management |  |  |  |  |  |
| 3 | Maintained airway patency using an appropriately sized airway adjunct when indicated |  |  |  |  |  |
| 4 | Applied the bag-mask device correctly to the patient’s face with an effective seal |  |  |  |  |  |
| 5 | Delivered ventilation at an appropriate rate and with visible chest rise |  |  |  |  |  |
| 6 | Performed airway-related duties in accordance with the assigned role |  |  |  |  |  |
| 7 | Demonstrated appropriate airway management technique during the scenario |  |  |  |  |  |
| 8 | Followed the Team Leader’s instructions appropriately |  |  |  |  |  |

6. Individual Performance Checklist: Nurse C

| No. | Assessment item | 5 Excellent | 4 Good | 3 Average | 2 Poor | 1 Very Poor |
| --- | --- | --- | --- | --- | --- | --- |
| 1 | Positioned appropriately around the patient’s bed |  |  |  |  |  |
| 2 | Correctly placed the CPR backboard under the patient’s chest |  |  |  |  |  |
| 3 | Performed chest compressions with appropriate rate, depth, and technique |  |  |  |  |  |
| 4 | Assessed the cardiac rhythm on the monitor when indicated |  |  |  |  |  |
| 5 | Performed defibrillation correctly when indicated and instructed by the Team Leader |  |  |  |  |  |
| 6 | Performed circulation-related duties in accordance with the assigned role |  |  |  |  |  |
| 7 | Demonstrated appropriate chest-compression technique during the scenario |  |  |  |  |  |
| 8 | Followed the Team Leader’s instructions appropriately |  |  |  |  |  |

7. Individual Performance Checklist: Nurse D

| No. | Assessment item | 5 Excellent | 4 Good | 3 Average | 2 Poor | 1 Very Poor |
| --- | --- | --- | --- | --- | --- | --- |
| 1 | Positioned appropriately around the patient’s bed |  |  |  |  |  |
| 2 | Obtained peripheral intravenous access using an appropriately sized cannula |  |  |  |  |  |
| 3 | Prepared medications and equipment required for ACLS according to the scenario |  |  |  |  |  |
| 4 | Administered indicated medications according to the Team Leader’s instructions and the patient’s condition |  |  |  |  |  |
| 5 | Recorded all administered medications, doses, and administration times accurately |  |  |  |  |  |
| 6 | Performed medication- and IV-related duties in accordance with the assigned role |  |  |  |  |  |
| 7 | Demonstrated appropriate medication preparation and administration technique during the scenario |  |  |  |  |  |
| 8 | Followed the Team Leader’s instructions appropriately |  |  |  |  |  |

8. Individual Performance Checklist: Nurse E

| No. | Assessment item | 5 Excellent | 4 Good | 3 Average | 2 Poor | 1 Very Poor |
| --- | --- | --- | --- | --- | --- | --- |
| 1 | Positioned appropriately around the patient’s bed |  |  |  |  |  |
| 2 | Assisted with defibrillation or chest compressions when required and instructed by the Team Leader |  |  |  |  |  |
| 3 | If family members were present, ensured that either the nurse or another available staff member remained with them and responded appropriately to their emotional needs |  |  |  |  |  |
| 4 | Facilitated family presence or contact at the earliest appropriate opportunity, in accordance with the clinical situation and local policy |  |  |  |  |  |
| 5 | If the patient died, treated the patient and family in a respectful manner consistent with cultural and ethical standards |  |  |  |  |  |
| 6 | Performed support-related duties in accordance with the assigned role |  |  |  |  |  |
| 7 | Demonstrated an appropriate supportive function within the resuscitation team |  |  |  |  |  |
| 8 | Followed the Team Leader’s instructions appropriately |  |  |  |  |  |

9. Individual Performance Checklist: Coordinator

| No. | Assessment item | 5 Excellent | 4 Good | 3 Average | 2 Poor | 1 Very Poor |
| --- | --- | --- | --- | --- | --- | --- |
| 1 | Positioned appropriately around the patient’s bed |  |  |  |  |  |
| 2 | Coordinated communication between ward personnel and the resuscitation team when necessary |  |  |  |  |  |
| 3 | Documented the resuscitation process continuously and in chronological sequence |  |  |  |  |  |
| 4 | Assisted with defibrillation or chest compressions when necessary and instructed by the Team Leader |  |  |  |  |  |
| 5 | Coordinated required arrangements for patient transfer when necessary |  |  |  |  |  |
| 6 | Performed coordination- and documentation-related duties in accordance with the assigned role |  |  |  |  |  |
| 7 | Demonstrated appropriate coordination and logistical support during the scenario |  |  |  |  |  |
| 8 | Followed the Team Leader’s instructions appropriately |  |  |  |  |  |

**Station 3. Post-Resuscitation Management**

Station Objective

To assess the team’s ability to provide immediate post-resuscitation care after return of spontaneous circulation (ROSC), including airway and ventilation management, hemodynamic monitoring, cardiac assessment, documentation, transfer coordination, temperature management as applicable, and family support.

Student Instruction

In this station, you are confronted with a scenario that requires you to act according to real clinical conditions and the assigned roles of the resuscitation team members.

A 29-year-old female patient developed cardiopulmonary arrest following intentional ingestion of tricyclic antidepressants and was transferred to the hospital by emergency medical services. After 20 minutes of cardiopulmonary resuscitation and three defibrillation shocks, return of spontaneous circulation is achieved, and a carotid pulse is palpable. The patient’s body temperature is 30 C. Her mother enters the resuscitation room and is visibly distressed. Demonstrate the actions you would take as members of the resuscitation team in the immediate post-resuscitation phase.

1. Required Equipment Checklist

| No. | Required equipment | Available | Not available |
| --- | --- | --- | --- |
| 1 | Hospital bed |  |  |
| 2 | Intubated resuscitation mannequin |  |  |
| 3 | Emergency trolley/crash cart with medications and equipment, including an adult bag-mask device, positioned beside the bed |  |  |
| 4 | Oxygen source (central or portable) |  |  |
| 5 | ECG machine |  |  |
| 6 | Monitoring equipment |  |  |
| 7 | Patient chart/medical record |  |  |
| 8 | Pens (2) |  |  |
| 9 | Bedside table in front of the patient’s bed |  |  |
| 10 | Presence of a family member/relative as part of the simulation |  |  |

2. Team Performance Checklist

| No. | Assessment item | 5 Excellent | 4 Good | 3 Average | 2 Poor | 1 Very Poor |
| --- | --- | --- | --- | --- | --- | --- |
| 1 | Performed appropriate checks to confirm and monitor endotracheal tube position |  |  |  |  |  |
| 2 | Ventilation was initiated and maintained at 10 breaths per minute |  |  |  |  |  |
| 3 | Monitoring was established to assess oxygenation after resuscitation |  |  |  |  |  |
| 4 | Blood pressure was assessed |  |  |  |  |  |
| 5 | A 12-lead ECG was obtained |  |  |  |  |  |
| 6 | Level of consciousness was assessed |  |  |  |  |  |
| 7 | Additional cardiac interventions were performed when indicated |  |  |  |  |  |
| 8 | Coordination with the intensive care unit was initiated when clinically appropriate |  |  |  |  |  |
| 9 | Post-resuscitation temperature management was initiated in accordance with the scenario and local protocol |  |  |  |  |  |
| 10 | One team member provided emotional support to the patient’s family member |  |  |  |  |  |

3. Overall Team Performance Checklist

| No. | Assessment item | 5 Acceptable | 3 Below expectations | 1 Unacceptable |
| --- | --- | --- | --- | --- |
| 1 | Coordination among team members was maintained during post-resuscitation management |  |  |  |
| 2 | Team members demonstrated sufficient speed and skill in managing the post-resuscitation phase |  |  |  |
| 3 | The team appropriately maintained the patient’s dignity and physical coverage during care |  |  |  |

4. Individual Performance Checklist: Team Leader

| No. | Assessment item | 5 Excellent | 4 Good | 3 Average | 2 Poor | 1 Very Poor |
| --- | --- | --- | --- | --- | --- | --- |
| 1 | Reassigned or confirmed team roles for the post-resuscitation phase |  |  |  |  |  |
| 2 | Positioned appropriately to oversee team activities and maintain situational awareness |  |  |  |  |  |
| 3 | Assessed the patient’s level of consciousness |  |  |  |  |  |
| 4 | Directed team members appropriately for ongoing ventilation and post-resuscitation management |  |  |  |  |  |
| 5 | Prioritized immediate post-ROSC assessments and interventions according to the patient’s condition |  |  |  |  |  |
| 6 | Coordinated decision-making regarding further cardiac evaluation and ongoing management |  |  |  |  |  |
| 7 | Ensured that patient transfer or escalation of care was initiated when clinically indicated |  |  |  |  |  |
| 8 | Communicated with the patient’s family member regarding events and the patient’s condition when appropriate |  |  |  |  |  |

5. Individual Performance Checklist: Nurse A&B

| No. | Assessment item | 5 Excellent | 4 Good | 3 Average | 2 Poor | 1 Very Poor |
| --- | --- | --- | --- | --- | --- | --- |
| 1 | Positioned appropriately around the patient’s bed for airway and ventilation management |  |  |  |  |  |
| 2 | Performed appropriate checks to confirm and monitor endotracheal tube position |  |  |  |  |  |
| 3 | Maintained airway patency and ensured airway-device stability during post-resuscitation care |  |  |  |  |  |
| 4 | Delivered ventilation at 10 breaths per minute |  |  |  |  |  |
| 5 | Assisted with monitoring oxygenation after resuscitation |  |  |  |  |  |
| 6 | Performed airway- and ventilation-related duties in accordance with the assigned role |  |  |  |  |  |
| 7 | Demonstrated appropriate airway and ventilation technique during the scenario |  |  |  |  |  |
| 8 | Followed the Team Leader’s instructions appropriately |  |  |  |  |  |

6. Individual Performance Checklist: Nurse C

| No. | Assessment item | 5 Excellent | 4 Good | 3 Average | 2 Poor | 1 Very Poor |
| --- | --- | --- | --- | --- | --- | --- |
| 1 | Positioned appropriately around the patient’s bed for circulation-related assessment and intervention |  |  |  |  |  |
| 2 | Measured and recorded the patient’s blood pressure |  |  |  |  |  |
| 3 | Obtained a 12-lead ECG |  |  |  |  |  |
| 4 | Assessed the patient’s cardiac rhythm and hemodynamic status when indicated |  |  |  |  |  |
| 5 | Performed indicated cardiac interventions in coordination with the Team Leader |  |  |  |  |  |
| 6 | Performed circulation-related duties in accordance with the assigned role |  |  |  |  |  |
| 7 | Demonstrated appropriate technique in monitoring and circulation-related support during the scenario |  |  |  |  |  |
| 8 | Followed the Team Leader’s instructions appropriately |  |  |  |  |  |

7. Individual Performance Checklist: Nurse D

| No. | Assessment item | 5 Excellent | 4 Good | 3 Average | 2 Poor | 1 Very Poor |
| --- | --- | --- | --- | --- | --- | --- |
| 1 | Positioned appropriately around the patient’s bed for medication and intravenous management |  |  |  |  |  |
| 2 | Assessed the patency and usability of the existing intravenous line or established access when required |  |  |  |  |  |
| 3 | Prepared and administered medications when indicated and instructed by the Team Leader |  |  |  |  |  |
| 4 | Counted used medication vials/ampoules and completed medication-related documentation |  |  |  |  |  |
| 5 | Assisted with temperature-management measures when indicated and in accordance with the scenario |  |  |  |  |  |
| 6 | Performed medication- and IV-related duties in accordance with the assigned role |  |  |  |  |  |
| 7 | Demonstrated appropriate medication preparation, administration, and post-administration follow-up during the scenario |  |  |  |  |  |
| 8 | Followed the Team Leader’s instructions appropriately |  |  |  |  |  |

8. Individual Performance Checklist: Nurse E

| No. | Assessment item | 5 Excellent | 4 Good | 3 Average | 2 Poor | 1 Very Poor |
| --- | --- | --- | --- | --- | --- | --- |
| 1 | Positioned appropriately to provide family support and auxiliary assistance without disrupting team performance |  |  |  |  |  |
| 2 | Communicated with the patient’s family member and responded appropriately to emotional and cultural needs |  |  |  |  |  |
| 3 | Facilitated family presence or contact at the earliest appropriate opportunity, in accordance with the clinical situation and local policy |  |  |  |  |  |
| 4 | Assisted the team with supportive bedside tasks when required and instructed |  |  |  |  |  |
| 5 | Helped maintain patient dignity, privacy, and physical coverage during care |  |  |  |  |  |
| 6 | If the patient died, treated the patient and family in a respectful manner consistent with cultural and ethical standards |  |  |  |  |  |
| 7 | Performed support-related duties in accordance with the assigned role |  |  |  |  |  |
| 8 | Followed the Team Leader’s instructions appropriately |  |  |  |  |  |

9. Individual Performance Checklist: Coordinator

| No. | Assessment item | 5 Excellent | 4 Good | 3 Average | 2 Poor | 1 Very Poor |
| --- | --- | --- | --- | --- | --- | --- |
| 1 | Positioned appropriately to facilitate coordination and documentation |  |  |  |  |  |
| 2 | Completed time-sequenced documentation of the resuscitation and post-resuscitation events accurately |  |  |  |  |  |
| 3 | Cross-checked medications, doses, and timing with the Team Leader or the administering nurse |  |  |  |  |  |
| 4 | Coordinated safe and timely transfer of the patient to the intensive care unit (ICU) |  |  |  |  |  |
| 5 | Facilitated communication between the resuscitation team, ward staff, and receiving ICU personnel |  |  |  |  |  |
| 6 | Provided logistical support for post-resuscitation tasks (e.g., organizing equipment cleaning/replenishment) |  |  |  |  |  |
| 7 | Performed coordination- and documentation-related duties in accordance with the assigned role |  |  |  |  |  |
| 8 | Followed the Team Leader’s instructions efficiently during the post-resuscitation phase |  |  |  |  |  |
